# A novel image registration approach via combining local features and geometric invariants

**DOI:** 10.1371/journal.pone.0190383

**Published:** 2018-01-02

**Authors:** Yan Lu, Kun Gao, Tinghua Zhang, Tingfa Xu

**Affiliations:** Key Lab of Photoelectronic Imaging Technology and System, Ministry of Education of China, Beijing Institute of Technology, Beijing, China; Worcester Polytechnic Institute, UNITED STATES

## Abstract

Image registration is widely used in many fields, but the adaptability of the existing methods is limited. This work proposes a novel image registration method with high precision for various complex applications. In this framework, the registration problem is divided into two stages. First, we detect and describe scale-invariant feature points using modified computer vision-oriented fast and rotated brief (ORB) algorithm, and a simple method to increase the performance of feature points matching is proposed. Second, we develop a new local constraint of rough selection according to the feature distances. Evidence shows that the existing matching techniques based on image features are insufficient for the images with sparse image details. Then, we propose a novel matching algorithm via geometric constraints, and establish local feature descriptions based on geometric invariances for the selected feature points. Subsequently, a new price function is constructed to evaluate the similarities between points and obtain exact matching pairs. Finally, we employ the progressive sample consensus method to remove wrong matches and calculate the space transform parameters. Experimental results on various complex image datasets verify that the proposed method is more robust and significantly reduces the rate of false matches while retaining more high-quality feature points.

## Introduction

Image registration is mainly for obtaining the transformation parameters between images taken at different times from different angles and sensors with translation, rotation, scaling and/or distortion to get the best match in the pixel layer [1]. Image registration is a fundamental issue for many computer vision technologies, such as image restoration, targeting, tracking, image stitching, image fusion, 3D reconstruction, and pattern recognition [2,3]. It is an important preliminary step to improve the accuracy and validity of the above problems.

Image registration techniques play an increasingly important role in various fields. For military applications, to improve precision strike capabilities, various images from different sensors, such as infrared, radar, and hyperspectral imaging, require high-precision registration [4,5]. The requirements are similar for civil use equipment for security monitoring, traffic control, wide field imaging and panoramic imaging [6,7]. In agriculture, image registration is also critical. For example, three-dimensional imaging devices, such as photonic mixer detectors, are used to capture image depth information. Those images are then matched with images from color cameras to more accurately record vegetation growth conditions [8]. Image registration is also an urgent issue for remote sensing applications, such as the investigation of geological disasters and the exploration of complex terrain. Image registration is often applied to optical images, synthetic aperture radar (SAR) images, and other multispectral images to achieve more surface feature information. Image registration has essential research value, especially in the medical field. Image registration technology is often used to obtain the exact location of organs and tissues from different image sources, such as X-ray computed tomography (CT), magnetic resonance imaging (MRI), single photon emission computed tomography (SPECT), and positron emission tomography (PET) [9,10], which enables reliable diagnosis and treatment. Therefore, research on image registration has important theoretical significance and practical application value.

**Fig 1:**
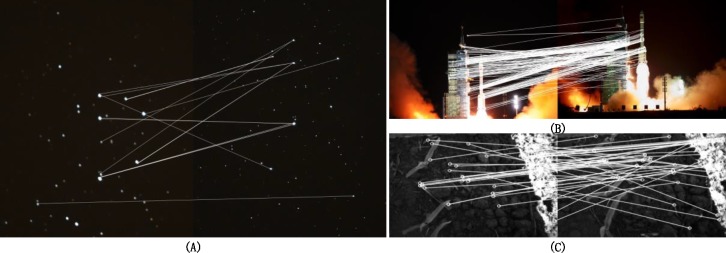
The mismatches in image registration.

The grayscale distribution, image details, and noise interference are all uncertain factors that challenge image registration. For instance, as in Fig 1A, many algorithms will fail to match the images with starry backgrounds since the images have a high dynamic range and lack texture features. The exposure time and the signal-to-noise ratio are also different. They all will lead to a high rate of false matches and even match failure. When the images have a large grayscale contrast and considerable noise interference, the mismatch rate will increase rapidly, as shown in Fig 1B. Therefore, the anti-interference ability and robustness of the algorithms are important factors in image registration. Moreover, accurate image registration is still challenged by images that have many similar details but small and detailed differences between the current and target images (see Fig 1C).

**Fig 2:**
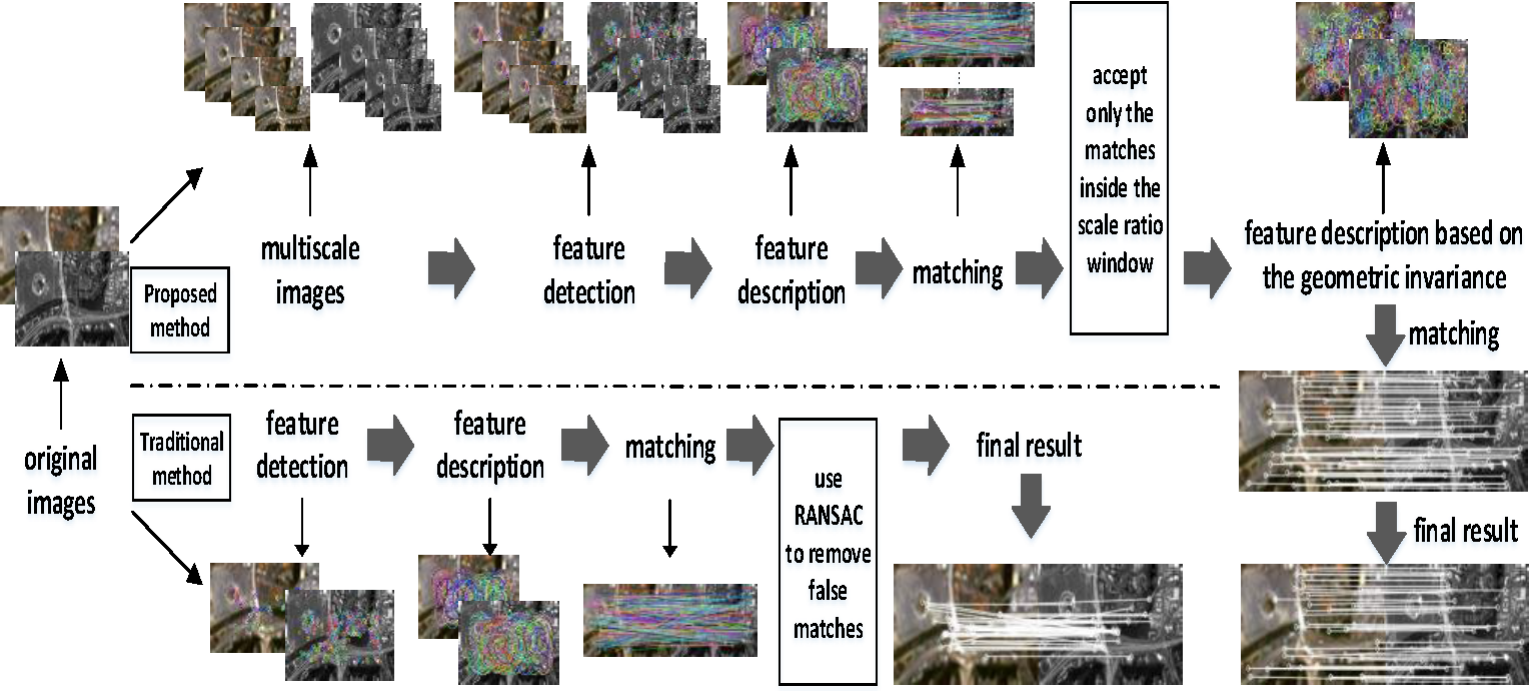
Proposed image registration framework.

Considering the problems above, this paper proposes a fast and robust image registration method, as shown in Fig 2. We utilize an improved feature detection and description method by modifying the computer vision-oriented fast and rotated brief (ORB) algorithms to provide scale invariance. Then, this approach is combined with the distribution of key points to form a robust method of feature selection and matching. For feature selection, a new distance window is set after considering the feature point location distribution and distance constraints. Then, high-quality feature points are extracted following the Hamming distance criterion and bidirectional matching constraint from the K nearest neighbor (KNN). We establish the feature description vector based on the geometric invariance, which differs from traditional matching methods. A cost function is constructed to evaluate the similarity of vectors in different dimensions and obtain better matched point pairs. Thus, even if there are relatively few feature points in images, we can still accurately register the images. Finally, space transformation parameters for image pairs are calculated after using the progressive sample consensus (PROSAC) algorithm to eliminate error matching; then, the final feature point matching relationship can be obtained.

## Related works

Previous researchers have proposed many excellent image registration methods, which can be classified as area- or feature-based. Regardless of the approach, the aim is to obtain the invariants from image pairs and find matching relations. Ideally, these invariants should not be affected by light, noise, or geometric deformation [11]. In recent years, methods based on local invariant feature points have become the major focus due to their superior performance.

Since the concept of corner detection was proposed by Moravec [12], many detection operators have been investigated, such as Harris, Shi-Tomasi, SUSAN, and others [13]. Lowe first proposed the scale invariant feature transformation (SIFT) algorithm [14]. The SIFT algorithm provided a huge improvement in accuracy for corner detection. It is robust to light changes, noise, and affine transformation and can achieve sub-pixel accuracy [15]. Bay et al. [16] improved SIFT’s efficiency and proposed the speed up robust feature (SURF) algorithm, which used Hessian matrices and distributed descriptors. SURF allows multiple images in a scale space to be processed simultaneously and does not require image subsampling. Therefore, it can effectively reduce descriptor dimensionality and significantly improve calculation speed while guaranteeing accuracy.

To apply the feature point detection algorithm in real time, Rosten and Drummond [17] proposed a new method called the features from accelerated segment test (FAST) algorithm. FAST compares surrounding pixels to obtain key points using machine learning. Therefore, it is simple, effective and easily ported to embedded systems [18]. Calonder et al. [19] proposed the BRIEF descriptor by comparing the PCA, LDA and other feature dimensional reduction methods. It reduces the time needed to generate feature descriptors by calculating and matching binary strings. Subsequently, FAST and BRIEF were redesigned by Rublee et al. They proposed the ORB algorithm at the 2011 IEEE International Conference on Computer Vision, which provided significant advantages in performance and speed [20].

In recent years, the registration technology based on image features has many applications. Zhang et al. [21] registered medical images by establishing a key feature model to describe the features and matching the corresponding points via a geometric constraint. Because the traditional methods were insufficient to achieve adequate results under different image deformations, Kahaki et al. [22] proposed an invariant feature matching method to overcome the limitations by measuring the dissimilarity of the features through the path based on eigenvector properties. Then, they achieved the registration of high resolution IKONOS satellite images. Li et al. [23] proposed an approach to robustly build key point mappings on multispectral images, and a similarity transformation was considered to account for the misalignment between two images. Lee et al. [24] proposed an application of the SIFT algorithm to stitch cervical-thoracic-lumbar (C-T-L) spine magnetic resonance (MR) images, and the results indicated that it can be improve diagnosis capabilities.

The local feature detection and matching methods described above have good real-time performance, noise immunity, robustness, and other positive characteristics, but image registration remains a challenging research topic [25]. Registration accuracy, reliability and computational time are three important characteristics that constrain universal registration methods in different circumstances. The traditional methods, such as SIFT or SURF, have excellent performance and high precision. However, when the images lack texture features, the feature points are difficult to extract and describe, and the matching result will be similar to Fig 1A. For images with rich textures, although it is possible to extract a large number of high-quality feature points, key point selection and precise matching still have many problems, particularly for images that are captured at different times, phases, or using different sensors, such as medical images. These images often have relative distortion, deformation, and/or uneven illumination. Traditional matching methods based on global or local features are limited by key point quantity and quality, which makes it difficult to guarantee precise results. In addition, it is difficult to describe the invariance of an image with many similar feature points by traditional methods. Therefore, we propose solving these problems by combining the modified local features and geometric invariants.

## Feature detection and description

Although many modified registration methods based on image features can theoretically enhance computational efficiency, ORB always performs better in complex scenarios [6]. It is fast, effective and accurate. Therefore, during feature detection and description, we improve the ORB algorithm to extract higher quality feature points to satisfy more complex applications.

In this step, we use the improved FAST-9 algorithm to detect features. A feature discriminant response function T is defined as

$$T=\sum_{i\forall (circle(p))}\left|G(i)-G(p)\right|>\xi \;(0<\xi <255),$$ (1)

where *G*(*p*) and *G*(*i*) are the grayscale values at *p* and its neighboring points and *ξ* is the threshold value. Here, we set the threshold to 40. When comparing the 16 neighbor points, if there are 9 consecutive points in the circular boundary of the neighborhood and their grayscale values are larger than *ξ*, it is judged to be a feature point. Then, the corner response function proposed by Harris was used to select from the identified feature points.

Then, the directions of feature points need to be calculated. For any of the feature points, the neighbor moments *M*_*pq*_ of the neighborhood pixels and the centroid of these moments *C* can be expressed as

$$M_{pq}=\sum_{x,y}x^{p}y^{q}I(x,y)\;\mathrm{and}\;C=\left(\frac{M_{10}}{M_{00}},\frac{M_{01}}{M_{00}}\right),$$ (2)

where *x*, *y* are the positions of feature points and the centroid can be calculated by *M*_*pq*_. The angle between the feature point and centroid is set as the dominant orientation, expressed as *α* = atan 2(*M*_01_,*M*_10_), where atan2 is the quadrant-aware version of arctan. These feature points provide directional invariance but are not scale invariant. This will be improved later.

After detecting the feature points, the improved BRIEF descriptor is used to describe these features. First, the point pairs are randomly generated in image patches. Let (*p*(*m*),*p*(*n*)) be the grayscale values for a point pair *p*, where each point pair corresponds to a binary string test *λ*.

$$\lambda (p;m,n)≔\{\begin{array}{l} 1\;\mathrm{if}\;p(m)<p(n)\\ 0\;\mathrm{otherwis}e \end{array}.$$ (3)

Then, *k* point pairs are randomly selected for generating a binary string. The feature descriptors *D*_*k*_ are expressed as

$$D_{k}(p)≔\sum_{1\leq i\leq k}2^{i-1}\lambda (p;m_{i},n_{i}).$$ (4)

These descriptors are based on the pixel values and are easily affected by noise. Therefore, a neighboring sub-window of feature points is defined, and the pixel value is replaced by comparing the gray-level integration of the sub-window. To ensure the descriptor has rotational invariance, *n* pairs of features are chosen at points (*x*_*i*_,*y*_*i*_) and form a matrix **S**.

$$S=\left(\begin{array}{c} x_{1},\;x_{2},\;\ldots ,x_{i},\;\ldots ,\;x_{n}\\ y_{1},\;y_{2},\;\ldots ,y_{i},\;\ldots ,\;y_{n} \end{array}\right)\;(i=1,2,\ldots ,n),$$ (5)

where (*x*_*i*_,*y*_*i*_) are the coordinates of the points. Then, using the dominant orientation and affine transformation matrix obtained in the feature detection stage, a new feature description matrix **S**' and descriptor *D*' can be calculated by rotating the affine transformation matrix such that

$$S'=\left[\begin{array}{cc} \cos\alpha & \sin\alpha \\ -\sin\alpha & \cos\alpha \end{array}\right]\cdot S,$$ (6)

$$D'(p,\alpha )≔D_{k}(p)|(x_{i},y_{i})\in S'.$$ (7)

Finally, using greedy search, 256 pixel pairs with minimum correlation can be found to describe the features.

To achieve scale invariance, traditional methods use the multiscale partitioning before feature detection, and then feature detection and extraction are separately performed. However, this often results in considerable mismatched features between low- and high-resolution images, which reduces the final matching rate. The SIFT algorithm also suffers from the same problem. Bastanlar et al. [26] proved this issue and proposed a preprocessing SIFT (PP-SIFT) solution.

In this work, we proposed an optimized method to reduce mismatches. The following processing steps are added to detect and match features of multiscale images.

Step 1: For images at a high resolution, we adopt a Gaussian low-pass filter and down sampling both horizontally and vertically.
Step 2: Apply ORB matching to images and plot the histogram of scale ratios.
Step 3: Form the histogram of scale differences and define a window |H_max_ ± *ω*| around the peak of histogram H_max_. Parameter *ω* is set between 0.20 and 0.35. The matches with scale differences outside this window are rejected.
Step 4: Extract the correct scale ratio (*d*) from the histogram as the mean of the most dominant Gaussian in the mixture.
Step 5: Accept only the matches with a scale ratio between 0.6*d* and 1.4*d*.

**Fig 3:**
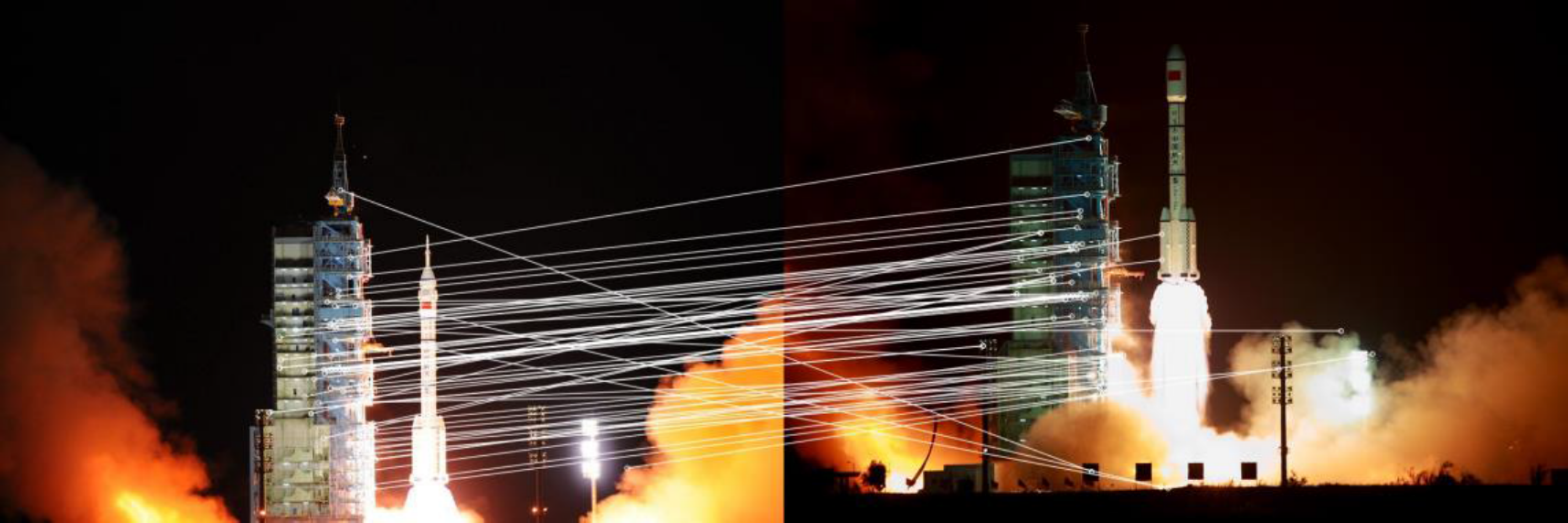
The matching under the proposed improved method.

Fig 1B is the matching result of the original ORB algorithm, and Fig 3 is the result of our improved method. Both methods remove false matches using the random sample consensus (RANSAC) [27,28]. The result shows that mismatches are significantly reduced, and a large number of correct matches remain after the optimization.

## Feature selection and matching method

The feature descriptor mentioned above is a binary string that provides increased storage and matching speed. The traditional matching method often utilizes the brute force (BF) [29] algorithm to match the feature points, which is followed by the RANSAC algorithm to eliminate mismatches. It is effective for normal scenes [30]. However, for complex applications, such as the matching of medical images or remotely sensed images, the generation of a large number of interference points can frequently occur and lead to a high mismatch rate [22]. Furthermore, when the feature points are sparse, it is difficult to guarantee an accurate matching [31]. To address this challenge, we propose new constraints of rough selection according to the distribution of feature points. We establish a new feature description vector and matching criterion based on geometrical relationships and employ the PROSAC algorithm for accurate matching.

### Constraint by feature distribution

Let *hd*_1_ and *hd*_2_ be the binary strings of feature descriptors for two images constructed by the ORB algorithm.

$$\{\begin{array}{c} hd_{1}=p_{1}\;p_{2}\;p_{3}\;\ldots \;p_{255}\\ hd_{2}=q_{1}\;q_{2}\;q_{3}\;\ldots \;q_{255} \end{array},$$ (8)

where *p* and *q* are the descriptors of two images. Then, the Hamming distances *D* of the image features is the XOR operation for the descriptors

$$D(hd_{1},hd_{2})=\sum_{i=0}^{255}p_{i}⊕q_{i}.$$ (9)

In traditional methods, feature points with Hamming distances smaller than a previously set threshold [32] *ε* are

$$Match_{j}=D_{j}\;(D_{j}<\varepsilon ),$$ (10)

where *D*_*j*_ are the distances of the *j*th match point pairs and *Match*_*j*_ are the selected matches. This method is applicable for images with relatively even distributions of features. However, if the image includes an energy-focused region or a region with an intensive feature distribution, it is difficult to define an appropriate threshold to avoid mismatches between nearby feature points. Since these points below the threshold are very similar, the image contains many low-quality feature points, especially in areas with strong noise interference.

**Fig 4:**
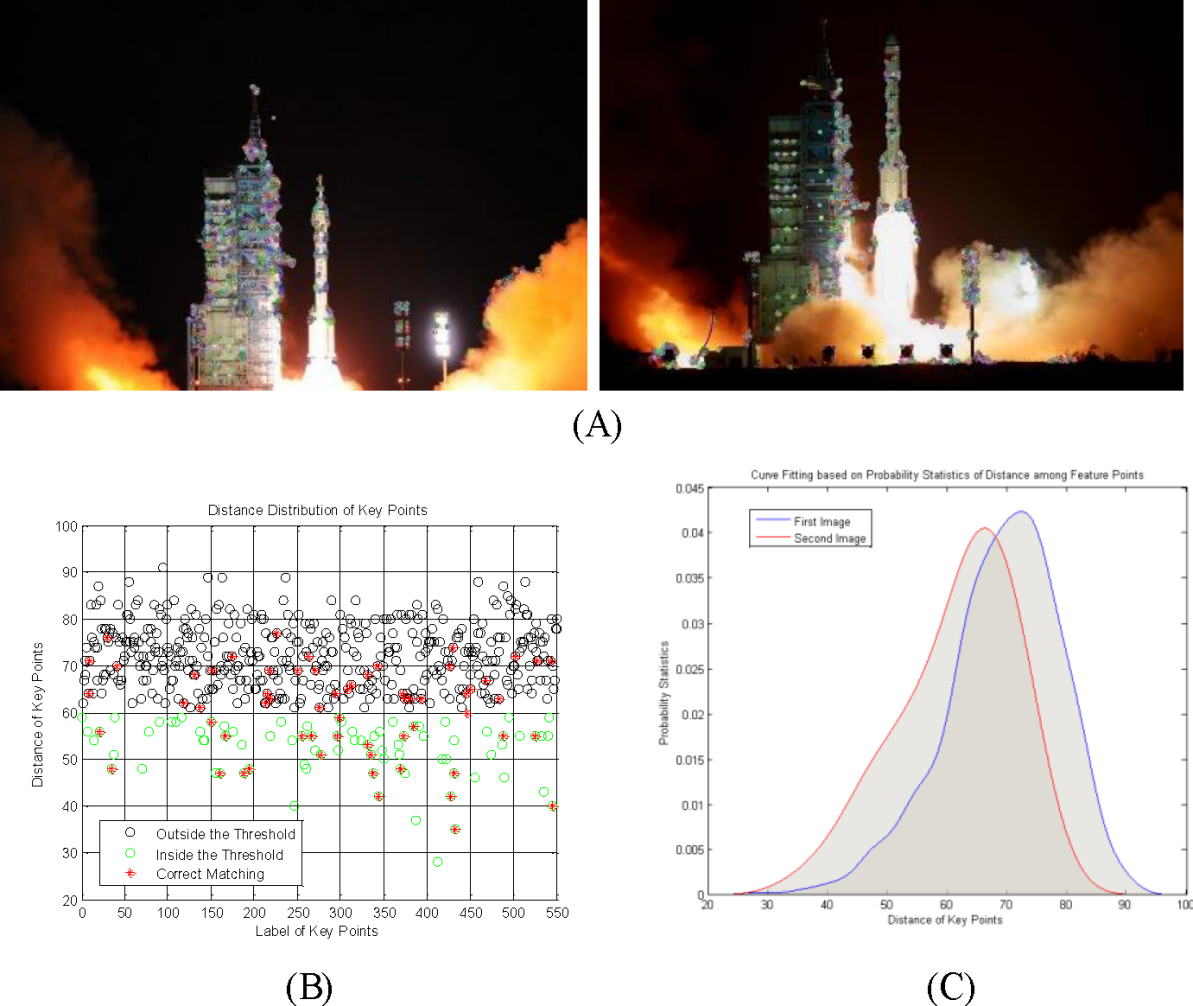
The distribution of feature points. (A) input images and the feature points; (B) the distance distribution of feature points; (C) curve fitting based on probability statistics of the distances.

Fig 4 is the distribution of feature points for two images. Fig 4A shows the detected feature points. The selection result is expressed by green circles when the threshold satisfies *ε* = 60, and the red stars (*) show the reliable matches, as shown in Fig 4B. The traditional method filters most of the low-quality feature points, but many correct matchings are also removed. The threshold parameter is unstable and unreliable as a selection standard.

Fig 4C shows the fitted curves based on the probability statistics of the feature point distances. It has a large overlap area around the mean with a corresponding high contact ratio, and most of the reliable feature points are distributed in the overlap area. Therefore, the mean centered constraint condition is set as a rough selection in this paper. We define the mean of root mean distance $\bar{D}$ as

$$\bar{D}=\sqrt{\frac{1}{n}\sum_{j}^{n}D_{j}}.$$ (11)

The selection window *R* is centered on $\bar{D}$ and defines the matching points to be retained.

$$\bar{D}-\;\varepsilon_{1}\leq R\leq \bar{D}+\varepsilon_{2}\;(\varepsilon_{1}>0,\varepsilon_{2}>0),$$ (12)

where *ε*_1_ and *ε*_2_ are the upper and lower limits of *R*, respectively, and can be modified according to the image feature distribution density. Here, we set *ε*_1_ to the medium value between the minimum distance and $\bar{D}$ and set *ε*_2_ to the medium value between the maximum distance and $\bar{D}$.

The distance constraint can remove significant errors and retain most of the reliable feature points, but it still needs further screening. KNN bilateral matching is employed in the following steps to select more reliable matching points.

Let *p*_*i*_ be a key point in the current frame, and let *p*_*j*1_ and *p*_*j*2_ be the two nearest matches of Hamming distance in the corresponding reference frame. Their distance are *D*(*p*_*i*_,*p*_*j*1_) and *D*(*p*_*i*_,*p*_*j*2_), which are optimal and sub-optimal, respectively. Similarly, there are two corresponding matching points for a key point in the reference frame with distances *D*(*p*_*j*_,*p*_*i*1_) and *D*(*p*_*j*_,*p*_*i*2_). There are also two candidate matching points based on the descriptor distance in another image.

The ratio of the optimal and sub-optimal value is used as the selection condition for the two images’ feature points. Two better quality sets of key points can be obtained using

R1'=D(pi,pj1)D(pi,pj2)<t,and (13)

R2'=D(pj,pi1)D(pj,pi2)<t. (14)

Here, we set *t* to 0.65 according to the experiments. Finally, matches that simultaneously satisfy both conditions are the respective optimal matches.

**Fig 5:**
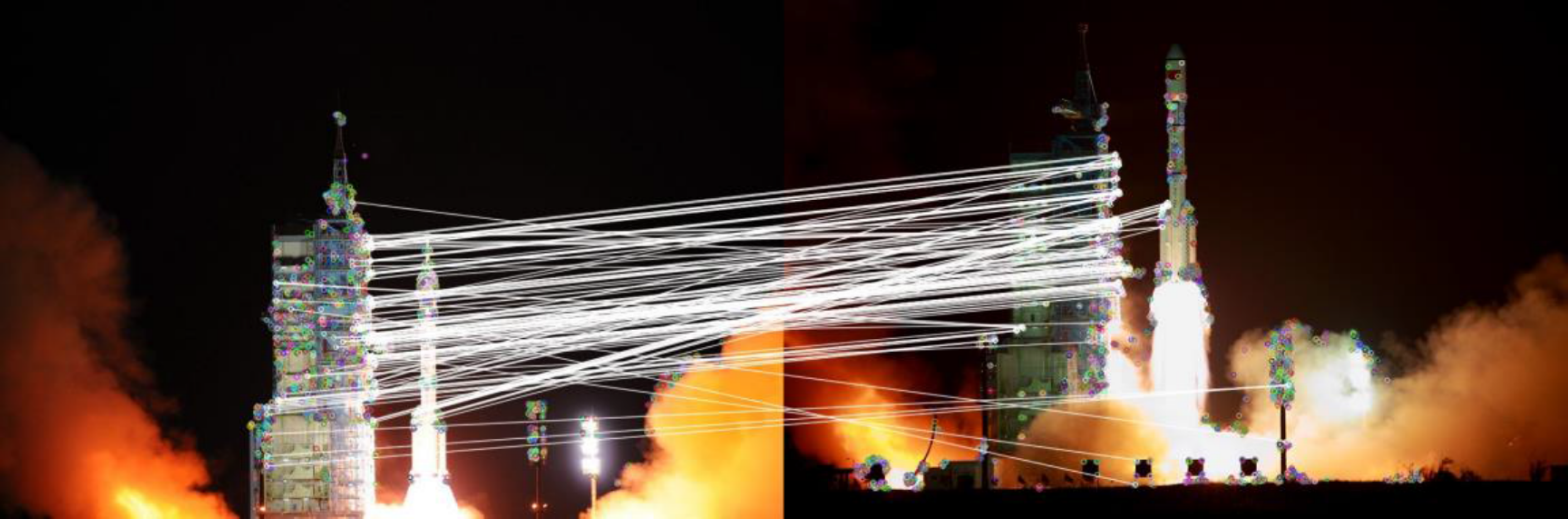
The matching result after the distance constraints.

After applying the distance constraint of the selection window and bilateral matching, many false matches are filtered without using RANSAC, as shown in Fig 5.

### A matching method based on geometric invariants

After the rough selection, the remaining feature points are more robust with higher quality. However, many mismatches may still exist for complicated situations. The main reason is that not all of the points identified by the Hamming distance match are correct matching pairs, and key point distance is only one factor considered for matching. The method has some limitations, especially when lacking texture and details or when there are few feature points [33]. It is also difficult to exactly match image pairs that have large deformations.

Therefore, this paper considers geometric invariance as a reliable matching factor, and a new method based on geometric invariants is proposed to provide further selection and matching of feature points. The geometric invariance was often used to describe the shape of objects, such as the shape context algorithm proposed by Belongie et al. [34]. It considers the object’s shape and contour in the image and makes full use of contextual information for image sequences. The algorithm uses the log polar histogram to describe the contour sampling distribution, and it is widely used for digital recognition, trademarks, and the like. Here, we briefly introduce the principles of this method and then propose our method.

Let the set **P** = {*p*_1_,*p*_2_,…,*p*_*m*_} with *m* sampling points describe an object’s shape. The log polar histogram *H*_*i*_(*m*) of the other *m*−1 sampling points is calculated as the shape contextual descriptor for each point.

$$H_{i}(m)=\#\{p_{j}\neq p_{i}:(p_{j}-p_{i})\in bin(m)\},$$ (15)

and the log polar transformation (LPT) can be expressed as

$$\rho =\log\sqrt{{\left(x-x_{0}\right)}^{2}+{\left(y-y_{0}\right)}^{2}},$$ (16)

$$\alpha =\arctan(\frac{y-y_{0}}{x-x_{0}}).$$ (17)

Then, divide *ρ* into five equal parts, divide *α* into twelve equal parts, and form *k* sections. Every point has its own distribution relative to the others, so the number of sampling points in each sub-sector domain can be used as the similarity criterion. Accordingly, using the matching cost function *F*_*i*,*j*_ between feature points *p*_*i*_ and *p*_*j*_, we get

$$F_{i,j}=\frac{1}{2}\sum_{m=1}^{k}\frac{{\left[H_{i}(m)-H_{j}(m)\right]}^{2}}{H_{i}(m)+H_{j}(m)}.$$ (18)

The feature point matching problem can be converted to match the weighted undirected bipartite graph. Finally, using the Hungary algorithm, we can find the optimal match and minimum cost value. Thus, the key points can be easily matched.

The feature of shape context can be easily extracted. The image scale and rotation transformation in the Cartesian coordinate system can be converted into the translation in the log coordinate system using LPT. Therefore, it has good scale and rotational invariance.

This algorithm has advantages for matching object shape, but the result may be affected by image noise and edge detection. Additionally, when objects are deformed, the matching accuracy and stability may be compromised [35]. The algorithm also requires that a point set must be the subset of a larger group, which is difficult to satisfy for image registration. Therefore, this paper utilizes the underlying theory of this method and proposes a new model to filter and match the feature points.

We define the matching point set obtained after coarse selection as **R** = {*p*_1_,*p*_2_,…,*p*_*n*_}. We calculate the distance from each key point *p*_*i*_ to the other *n* − 1 key points *p*_*j*_ without dividing the sharp histogram. The distances *D*_*i*_(*n*) between these points can be expressed as

$$D_{i}(n)=\|p_{i}-p_{j}{\|}_{2},\;p_{j}\in R,\;i\neq j.$$ (19)

Assuming that *k* is the number of feature points, a feature description matrix with *k* × (*k* − 1) dimensions can be obtained.

If the dimension is different between two images, the similarity among feature vectors cannot be measured by Eq (18). Therefore, an improved descriptive model is proposed. Let *m* and *n* be the number of feature points in the two images, with the feature vectors

$$\{\begin{array}{c} D_{i}=(D_{i1},D_{i2},\ldots ,D_{im-1})\;\\ D_{j}=(D_{j1},D_{j2},\ldots ,D_{jn-1}) \end{array}.$$ (20)

Then, a new matching cost function *F*_*i*,*j*_ that considers the feature point distances is defined as

$$F_{i,j}=\sum_{s=1}^{m-1}\sum_{t=1}^{n-1}\exp(\frac{{\left[D_{i}(s)-D_{j}(t)\right]}^{2}}{\sigma^{2}}),$$ (21)

where *D*_*i*_(*s*) and *D*_*j*_(*t*) are the feature vectors of the current image and target image, respectively. *σ* is the controllable distance error threshold set it to 1 in this paper.

**Fig 6:**
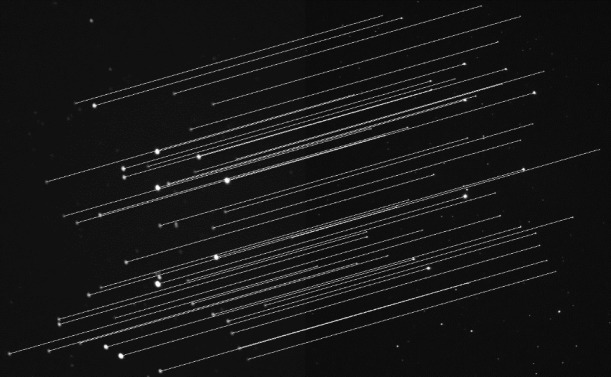
The matching result of Fig 1A by our proposed method.

Finally, we construct the binary search trees for every key point according to the cost function. Then, we calculate the ratio of the previous *K* nodes and compare them with threshold *T* to judge whether they are an acceptable matching point pair.

$$R_{m}=\frac{F_{i,j}(max)}{F_{i,j}(m)}>T,\;(m=1,2,\ldots ,K),$$ (22)

where *F*_*i*,*j*_(*m*) is the matching cost function and *F*_*i*,*j*_(*max*) is the maximum matching. We set the parameter *T* to 0.8 in this paper. This method considers location distribution and geometrical relationships among the feature points. Even in the case of few feature points, such as Fig 1A, it can achieve highly accurate matching, as shown in Fig 6.

To make the matching method more robust, we employ the progressive sample consensus algorithm that improves the RANSAC to remove outliers. The RANSAC algorithm does not deal well with the situation in which the number of mismatched pairs is too large in the proportion to the total matched pair, and it may fail, as shown in Fig 1C. The progressive sample consensus algorithm uses a data subset with a high matching rate as the sample set to estimate fitting [27]. It realizes rapid convergence and can deal with high mismatching. Following the methods discussed above, we can select feature points.

Then, the matched points can be easily sorted according to quality. The steps to remove the final mismatched pairs in this paper are as follows.

1. Feature points, identified following the procedures discussed above, are sorted in descending order by matched degree.
2. Set the sampling frequency, sample set *λ*, and sample size *η*. Each sample includes the coordinates of one feature point. The initial sample size *η* = 4 is the minimum to estimate the transformation matrix. After each loop, the sample size increases to *η* = *η* + 1.
3. Determine the initial 4 sample set. Three matching point pairs are randomly chosen from *λ*, and then the *η*th matching point pair from *λ* is added to constitute the initial sample set. The transformation matrix **M** transforms the coordinate from (*u*',*v*') into (*u*,*v*) and can be estimated from the initial sample set,

$$\left[\begin{array}{c} u\\ v\\ 1 \end{array}\right]=M\left[\begin{array}{c} u'\\ v'\\ 1 \end{array}\right]=\left[\begin{array}{ccc} \cos\alpha & -\sin\alpha & \Delta u\\ \sin\alpha & \cos\alpha & \Delta v\\ 0 & 0 & 0 \end{array}\right]\left[\begin{array}{c} u'\\ v'\\ 1 \end{array}\right].$$ (23)

4) Judge whether the initial matching point pair can satisfy the following two sampling termination conditions. If the conditions are not met, repeat step 2. Otherwise, exit the loop.
① The ratio between the number of inner points and the total points is larger than the error threshold, *ξ*. If *p* and *p*' are a matching point pair, the condition to be an inner point is ‖**M***p*−*p*'‖^2^ ≤ *ξ*.
② The rate of increase of inner points should be less than the increase threshold, *ξ*'. In other words, the number of inner points should increase slowly after a certain number of samplings.

## Experimental procedure and results

**Fig 7:**
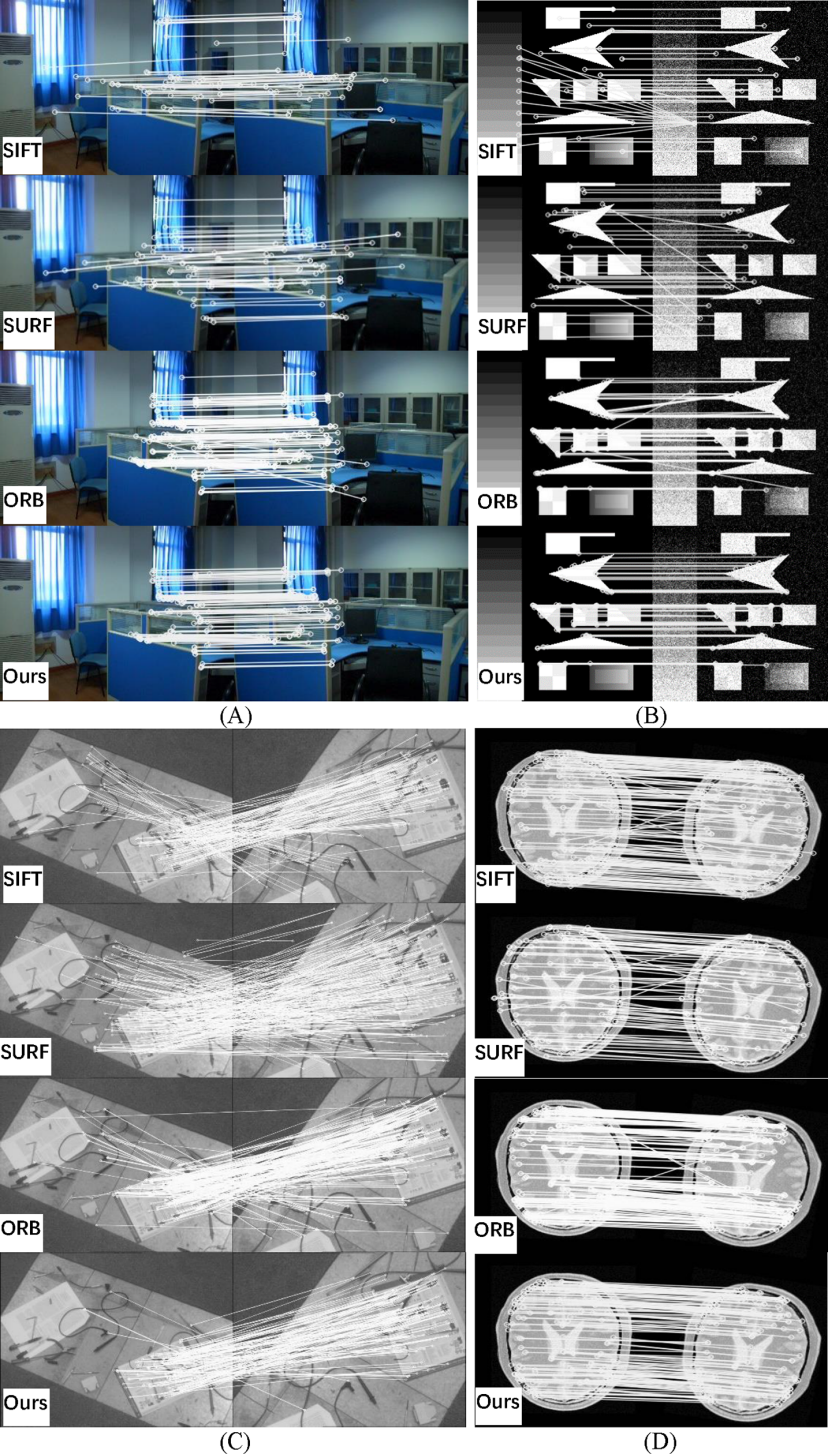
Visual matching between SIFT, SURF, ORB and the proposed method for typical images. (A) indoor; (B) noise; (C) remote sensing; and (D) medical.

**Fig 8:**
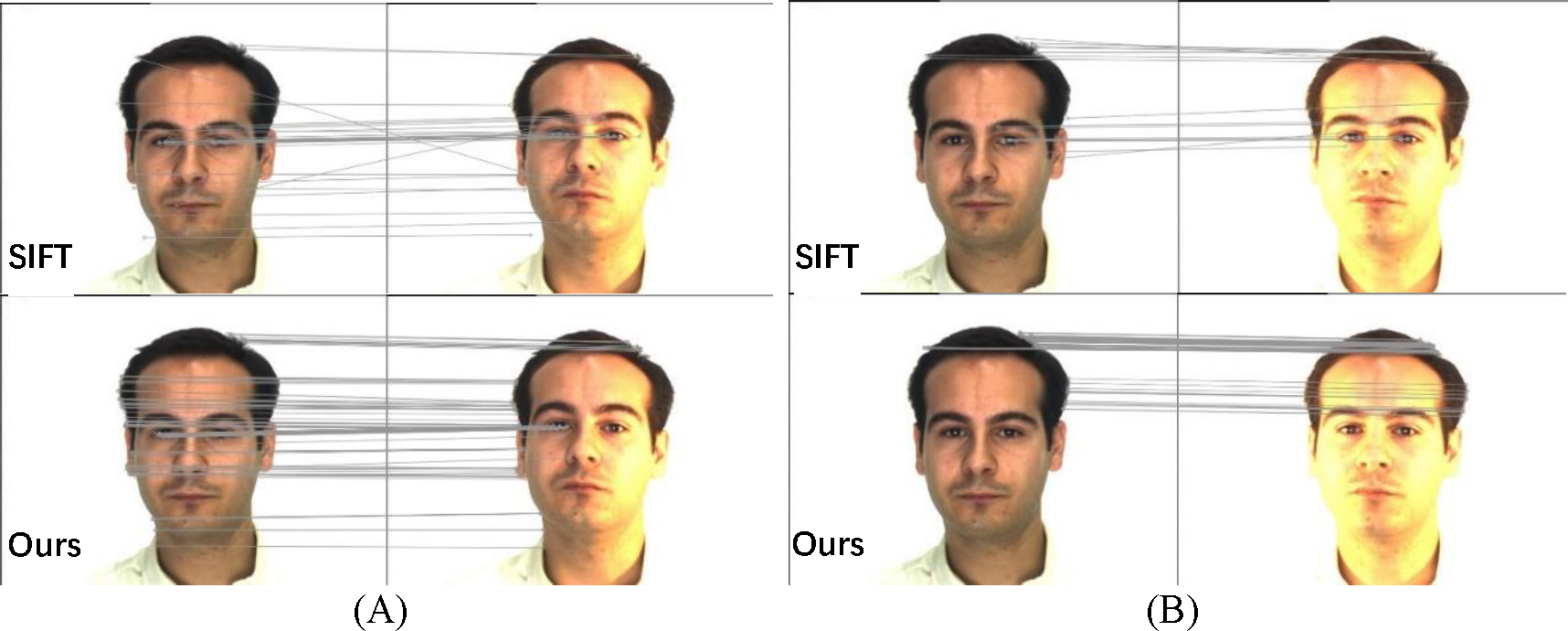
Visual matching between SIFT and the proposed method for images with more difficult registrations. (A) small illumination variation; (B) strong illumination variation.

**Fig 9:**
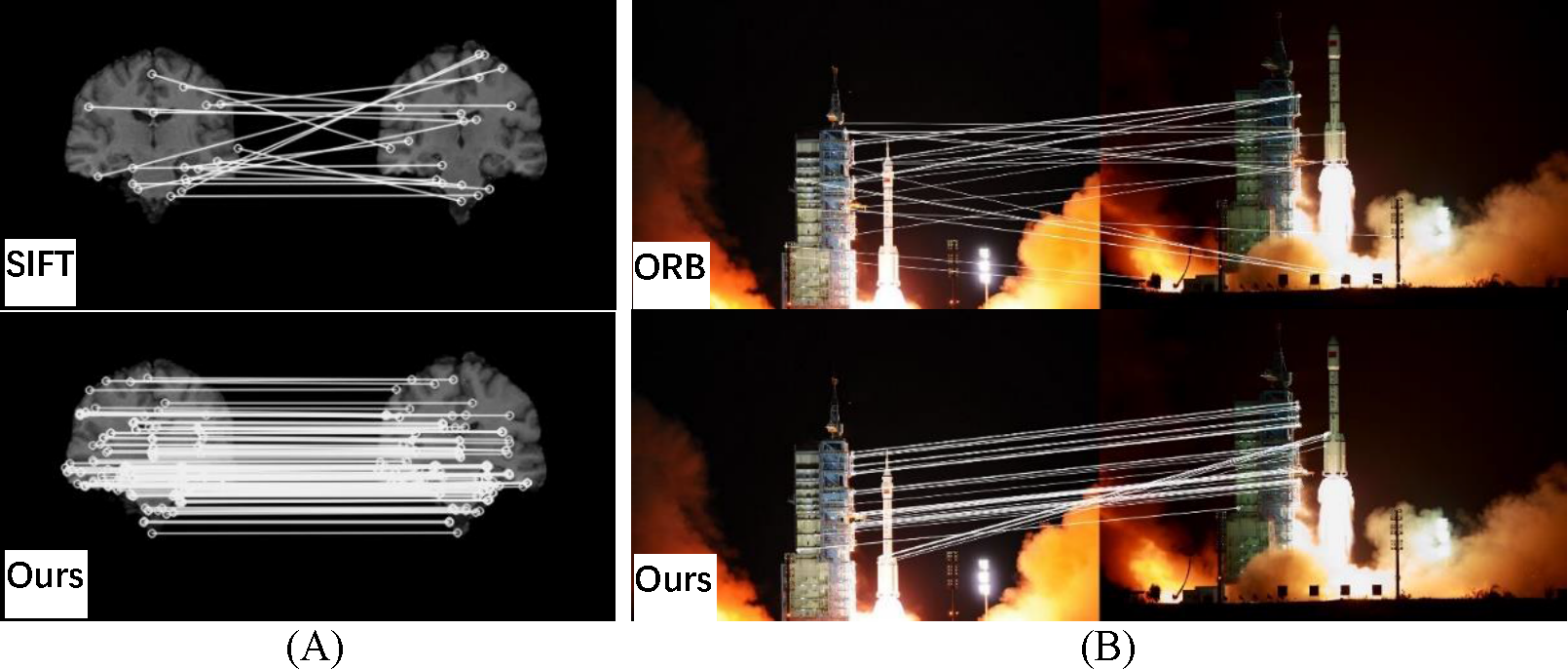
Visual matching among SIFT, ORB and the proposed method for more challenging images. (A) medical; (B) launch of the Tiangong rocket.

**Fig 10:**
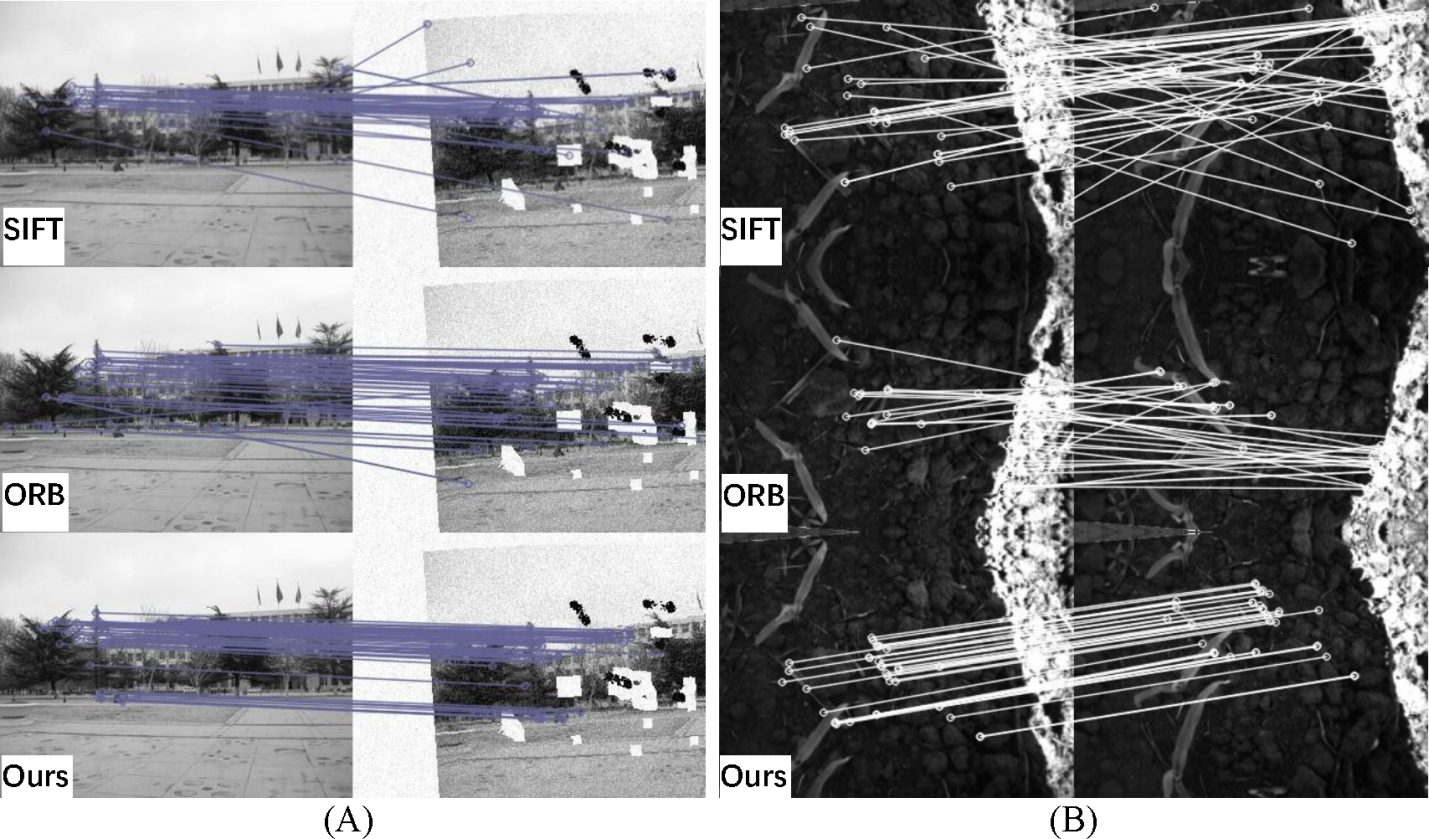
Visual matching for SIFT, ORB, and the proposed method. (A) low SNR (signal-to-noise ratio); (B) high dynamic range.

**Fig 11:**
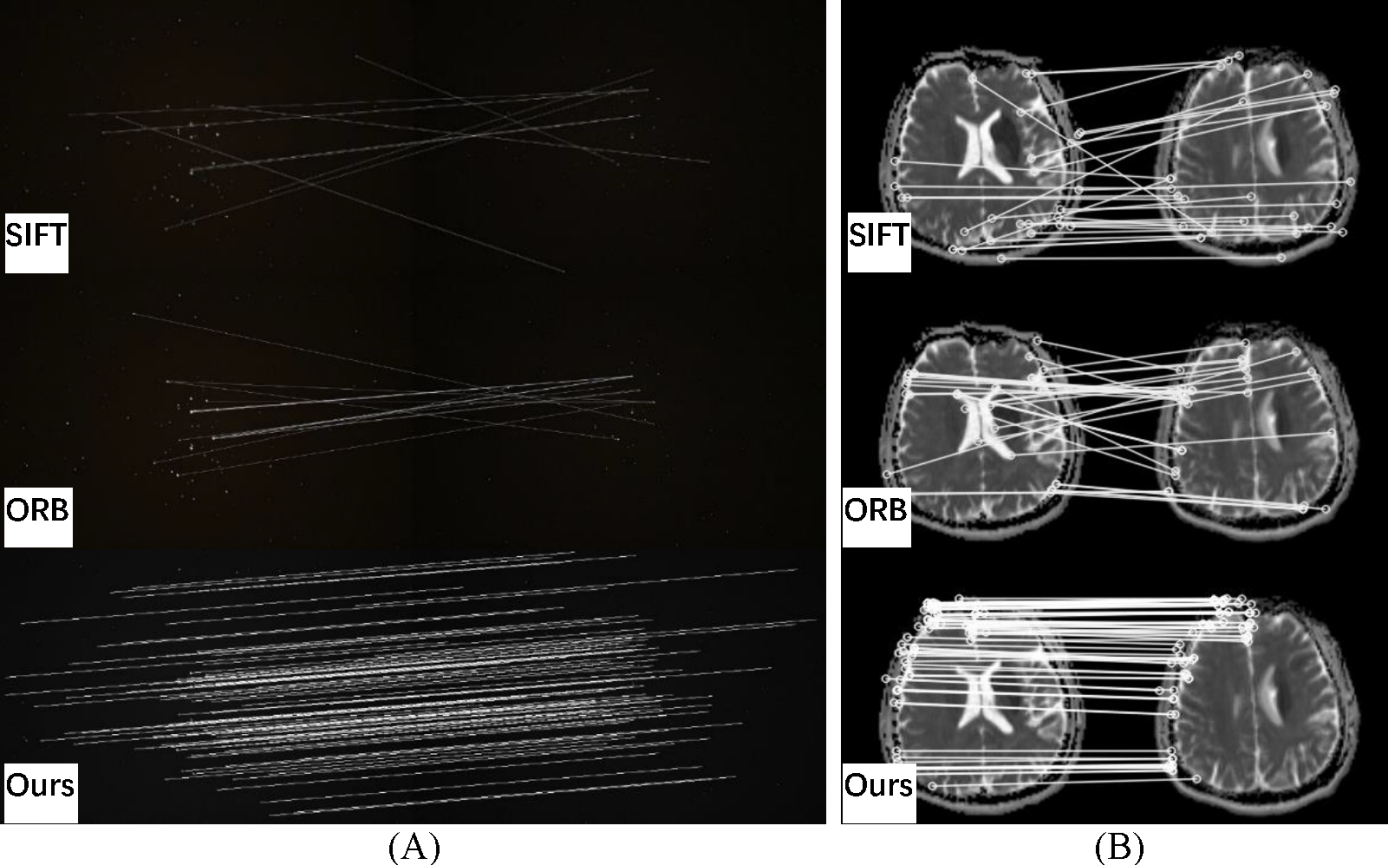
Visual matching for SIFT, ORB, and the proposed method. (A) Pleiades; (B) medical.

The SIFT algorithm is often used in image registration and has better performance than many others methods. Here, we compare the SIFT algorithm to the BF algorithm employed to match the feature points and the RANSAC algorithm employed to eliminate mismatches. We show the final visual matching results. To examine the performance and robustness of our proposed method in various situations, test images were chosen with different resolutions and different applications. Fig 7, Fig 8, Fig 9, Fig 10 and Fig 11B are from the public image database. The other images were newly taken using a digital camera. Visual qualitative contrasts (including the connecting line between matching point pairs in two images) and quantitative comparisons (involving the different parameters and calculation results) were performed.

### Qualitative comparison results

Fig 7 shows a visual comparison of typical images. Methods such as SIFT, SURF, ORB and the proposed method are compared in each group. The SIFT algorithm combined with RANSAC has good performance, but there are still some significant mistakes that are difficult to remove by using RANSAC. In our experiment of other registration algorithms, such as SURF and ORB, the results have similar outcomes to SIFT. All methods perform well when faced with a rotation of over 30 degrees, as shown in Fig 7C. The proposed method is significantly more accurate, and it provides a more uniform key point distribution due to the multi-layer selection.

Fig 8 shows the outcomes for more challenging images with excess exposure of the face. The images in Fig 8A and 8B are from the public Purdue AR face image database [36]. The SIFT algorithm produces many mismatches, whereas the proposed method has more reliable matching. Large numbers of correct matches are produced for varying illumination.

Fig 9 shows increasingly complex images. The images in Fig 9A have many similar details with a single background. This makes a large number of feature points difficult to match, and most of them are removed by RANSAC. The SIFT algorithm provides many mismatches, while the proposed method can precisely complete registration.

Fig 9B images are from the launch of the Tiangong rocket at different times. They have rich details, but the image content changes significantly. There are also large differences in brightness between the tail flame of the rocket and the dark background. The changing flight altitude and thick smoke can also seriously affect registration. Fig 1A and the upper image in Fig 9B are the result of SIFT and ORB, respectively. It shows that both the SIFT and ORB algorithms fail to accurately register this case. In contrast, the proposed method still has a large number of high quality matches.

In summary, the proposed method shows excellent performance in feature extraction, selection and matching, even for very complex images.

### Quantitative comparison results

A further four groups of images were analyzed using SIFT and ORB with BF. Then, RANSAC was used to remove feature selection mismatches. These images correspond to different situations, including low signal-to-noise ratio, high dynamic range, lack of textures and medical images with local similarity. The number of matching points, the points after screening, the number of false matches and the matching rate were calculated and compared. Visible results and the quantitative comparisons for these test images are shown in Figs 10 and 11 and Table 1, respectively.

**Table 1:** Comparative metrics for the images in Figs 10 and 11.

Image pair	Image type	Resolution	Methods	Initial matching points	Matching points after screening	Mismatches after screening	Matching rate
Fig 10A	low SNR ratio images	320×240	SIFT, RANSAC	468	38	16	57.89%
			ORB, RANSAC	511	51	23	54.90%
			**Proposed**	**556**	**73**	**7**	**90.41%**
Fig 10B	high dynamic range images	320×240	SIFT, RANSAC	569	55	34	38.18%
			ORB, RANSAC	391	35	27	22.86%
			**Proposed**	**771**	**68**	**2**	**97.06%**
Fig 11B	Pleiades images	749×249	SIFT, RANSAC	387	15	15	0.00%
			ORB, RANSAC	499	19	14	26.32%
			**Proposed**	**486**	**97**	**0**	**100.00%**
Fig 11B	medical images	230×230	SIFT, RANSAC	450	28	17	39.29%
			ORB, RANSAC	411	34	22	35.29%
			**Proposed**	**622**	**150**	**14**	**90.67%**

Fig 10A images show strong noise interference and low image quality. Both the SIFT and ORB algorithms have nearly half the error matches after screening by RANSAC. The proposed method removes the low reliability matching points, thus significantly reducing the final mismatch rate.

The images in Fig 10B have a large dynamic range and many similar features, but they lack details. Although a large number of feature points is extracted by SIFT and ORB, the methods still suffer a high mismatch rate, and the algorithms are almost invalid. The proposed algorithm has outstanding performance in this situation, with a final matching rate of 97.06%, compared to 38.18% for the SIFT algorithm and 22.86% for the ORB algorithm.

Fig 11A shows the registration result of the Pleiades images. The exposure time of reference image (left) is 0.5 s, and the ISO is 6400. The exposure time of the target image (right) is 0.25 s, and the ISO is 12800. The star images have almost no texture but a high dynamic range. The results show that the SIFT and ORB both fail to properly register this case, whereas the proposed method shows outstanding performance with a 100% matching rate. The images in Fig 11B were taken at different times with many similar details, and they have local deformation, which often occurs in medical images. The SIFT and ORB algorithms have a large number of false matches, so the RANSAC algorithm cannot work well, which can lead to a matching failure. The proposed method has a larger number of correct matches, with a matching rate of 90.67%.

The average time consumption of the different methods is shown in Table 2. Feature matching is the most time consuming aspect. If *N*_1_ and *N*_2_ are the number of feature points in two images, then the complexity of BF matching is *O*(*N*_1_ * *N*_2_). Therefore, the number of key points involved in matching will directly impact the algorithm’s efficiency. In general, in the step of feature detection, the number of key points is similar between SIFT/ORB and the proposed method, but the proposed method’s processing can be up to 10 times faster than SIFT. In addition, the proposed method’s processing speed is similar to that of ORB, but the number of correct matches is far greater than that of ORB.

**Table 2:** Time consumption.

Methods	Time to analyze the image group (s)				Average time (s)
	Fig 10A	Fig 10B	Fig 11A	Fig 11B	
SIFT	3.321	1.689	29.886	2.958	9.464
ORB	0.283	0.539	1.357	0.263	0.611
**Ours**	**0.346**	**0.591**	**1.557**	**0.322**	**0.704**

## Conclusion and future work

Considering the limitations of traditional methods, this paper proposed a fast and robust image registration approach based on local features and geometric invariants. In the step of feature detection and description, we proposed an improved method of the ORB algorithm. The proposed method is scale invariant and produces more higher quality feature points. Then, we improved the removal of mismatches by combining it with the distribution of key points. A new distance constraint window is set according to the distribution of feature points, and the bidirectional matching constraint from the K nearest neighbor is utilized to extract higher quality feature points.

To further improve the method’s adaptability and robustness and obtain the optimum matching point pairs, we proposed a novel geometric constraints matching algorithm with a new feature description vector based on the geometric invariance and a new cost function. Appropriate selection criteria were established to remove unreliable matches, and we integrated the PROSAC algorithm to further remove false matches. Thus, even with complex situations, we are still able to accurately register the images.

The experimental results show that our proposed registration method has superior adaptability and stronger robustness in terms of increasing the number of reliable key points and reducing the mismatch rate compared to the SIFT and ORB algorithms.

Future work may include improving the efficiency and real-time applications. The proposed method has fast matching speed using binary string descriptors, so parallel processing may be considered to allow higher resolution images to be processed in real time.
